# MT1-MMP regulates urothelial cell invasion via transcriptional regulation of Dickkopf-3

**DOI:** 10.1038/sj.bjc.6604513

**Published:** 2008-07-29

**Authors:** K Saeb-Parsy, A Veerakumarasivam, M J Wallard, N Thorne, Y Kawano, G Murphy, D E Neal, I G Mills, J D Kelly

**Affiliations:** 1 Department of Uro-Oncology, CRUK Cambridge Research Institute, Cambridge CB2 0RE, UK; 2 Huchison/MRC Research Centre, Cambridge CB2 0XZ, UK; 3 Department of Bioinformatics, CRUK Cambridge Research Institute, Cambridge CB2 0RE, UK; 4 Department of Oncology, Imperial College London, London W12 0NN, UK; 5 Department of Tumour Micro-Environment, CRUK Cambridge Research Institute, Cambridge CB2 0RE, UK

**Keywords:** MT1-MMP, invasion, DKK3, urothelial cell carcinoma, transcription

## Abstract

Membrane type-1 matrix metalloproteinase (MT1-MMP) is a zinc-binding endopeptidase, which plays a crucial role in tumour growth, invasion and metastasis. We have shown previously that MT1-MMP has higher expression levels in the human urothelial cell carcinoma (UCC) tissue. We show here that siRNA against MT1-MMP blocks invasion in UCC cell lines. Invasion is also blocked by broad-spectrum protease and MMP inhibitors including tissue inhibitor of metalloproteinase-1 and -2. Membrane type-1-MMP can also regulate transcription. We have used expression arrays to identify genes that are differentially transcribed when siRNA is used to suppress MT1-MMP expression. Upon MT1-MMP knockdown, Dickkopf-3 (DKK3) expression was highly upregulated. The stability of DKK3 mRNA was unaffected under these conditions, suggesting transcriptional regulation of DKK3 by MT1-MMP. Dickkopf-3 has been previously shown to inhibit invasion. We confirm that the overexpression of DKK3 leads to decreased invasive potential as well as delayed wound healing. We show for the first time that the effects of MT1-MMP on cell invasion are mediated in part through changes in DKK3 gene transcription.

Matrix metalloproteinases (MMPs) are a group of zinc-dependent endopeptidases that regulate the pericellular microenvironment and play an important role in development and physiological regulation of the extracellular environment (Woessner, 1991; Nagase *et al*, 2006). Membrane type-1 MMP (MT1-MMP) was the first member of the membrane-bound MMPs to be identified, and it plays a crucial role in tumour growth, angiogenesis, invasion and, ultimately, development of metastasis (Sato *et al*, 1994; Zhou *et al*, 2000; Sabeh *et al*, 2004; Itoh and Seiki, 2006).

Membrane type-1-MMP enhances degradation of collagen IV, a major component of the basement membrane, by forming a complex with tissue inhibitor of metalloproteinase-2 (TIMP-2) to activate pro-MMP-2 (Seiki, 2003). The process of substrate activation is a well-characterised function of MT1-MMP, and other substrates requiring cleavage and shedding for activation, including MMP13, CD44 and *α*-v-*β*-3, have been identified (Knauper *et al*, 1996; Kajita *et al*, 2001; Beauvais and Rapraeger, 2003, 2004; Endo *et al*, 2003; Nagano and Saya, 2004). Recently the functional role of MT1-MMP has been extended beyond sheddase activity; MT1-MMP induces VEGFA and Smad1 and the suggested mechanism is transcriptional regulation (Sounni *et al*, 2004; Freudenberg and Chen, 2007). Induction of VEGFA appears to be through the activation of src kinase pathway, and the presence of catalytic and cytoplasmic domains are crucial in this process, which suggests that MT1-MMP is a transcriptional regulator of multiple targets (Sounni *et al*, 2004).

Bladder cancer or urothelial cell carcinoma (UCC) is the fifth common malignancy in the United Kingdom (Cancer Research UK (CRUK) cancer statistic 2003), and it is anticipated that in 2007 over 67 160 new cases would be diagnosed in the United States of America (Jemal *et al*, 2007). We have previously demonstrated that of the MMPs, MT1-MMP is highly expressed in UCC, localising to both the epithelial and stromal compartments and associated with stage and grade progression (Wallard *et al*, 2006). Expression of MT1-MMP is highest in tumours invading the lamina propria (pT1) and detrusor muscle (>pT2). This expression is in keeping with the propensity for up to 20% of pT1 tumours to progress to muscle invasive disease, and for up to 50% of muscle invasive tumours to metastasis, within 2 years (Torti and Lum, 1984).

In this study, we explored the potential downstream events following targeted manipulation of MT1-MMP in UCC cell lines. We employed expression microarray to highlight changes in the expression of genes following suppression of MT1-MMP. Among candidate genes, we identified Dickkopf-3 (DKK3) as a putative target, which appears to be transcriptionally suppressed by MT1-MMP and by itself is a potent regulator of cell invasion. Dickkopf-3, also known as reduced expression in immortalised cells (REIC), is a divergent member of a group of four secreted proteins, and emerging evidence suggests that it functions as a tumour suppressor to inhibit cell growth and motility (Hsieh *et al*, 2004; Kawano *et al*, 2006). Dickkopf-3 may interact to inhibit wnt-signalling pathways (Hoang *et al*, 2004), and its loss in UCC and other cancers corresponds to a tumour suppressor effect (Nozaki *et al*, 2001; Kurose *et al*, 2004; Kawano *et al*, 2006; Urakami *et al*, 2006). In support of an extended role for MT1-MMP, the results of this study offer further evidence of interaction with other targets, specifically with negative regulation of the antimigratory tumour suppressor gene DKK3 in UCC.

## Materials and methods

### Cell lines and culture

Urothelial cell carcinoma cell lines RT112, 253JBV and EJ28 were sourced from CRUK. UMUC3 and HT137 were sourced from the American Type Culture Collection. UMUC3, 253JBV and 253JBV were grown in RPMI 1640 supplemented with 10% foetal bovine serum. The remainder of the cell lines were grown in DMEM supplemented with 10% foetal bovine serum. Cells were maintained at 37°C in a humidified incubator containing 95% air and 5% CO_2_.

### Constructs

pCS2-hDKK3 and pCDNA-T05 empty vectors were kind gifts from Y Kawano (Imperial college, London, UK) and J Girling (Cambridge Research Institute, Cambridge, UK), respectively. FuGENE 6 (Roche, Welwyn Garden City, UK) was used for transfection.

### Cell culture treatments

Cells were treated for 24 h with synthetic and physiological protease inhibitors: aprotinin (10 *μ*g ml^−1^), BB94 (5 *μ*M), TIMP-1 (500 nM) and TIMP-2 (500 nM) in growth media. For functional studies, cells were pretreated as above and maintained in media with the inhibitor throughout the experiment. For invasion assays, inhibitors at the above-mentioned concentrations were also added to the Matrigel™ mix. Aprotinin, BB94 and purified recombinant TIMP-1 and TIMP-2 were kind gifts from Professor Murphy.

### siRNA transfection

siGENOME Individual Duplex Human MT1-MMP siRNA and si*CONTROL* non-targeting siRNA were purchased from Dharmacon (Chicago, IL, USA) and used at 20 *μ*M. Opti-MEM™ transfection media and oligofectamine™ (both from Invitrogen, Paisley, UK) were used to transfect the cells once they reached 50% confluency. Knockdown was assessed by both quantitative reverse-transcriptase polymerase chain reaction (qRT-PCR) and western blot analysis. To block transcription, 24 h following the knockdown of MT1-MMP, cells were incubated in serum-free media for 12 h and actinomycin D (10 *μ*g ml^−1^) was added. Cells were harvested at time 0 as well as time points 1, 2, 4 and 8 h following the addition of actinomycin D, and qRT-PCR analysis was performed for DKK3 and MT1-MMP mRNA expression.

### Isolation of secreted DKK3 from media

Following knockdown of MT1-MMP, cells were grown in opti-MEM (Invitrogen). Media were concentrated using Vivaspin column (pore size 1 × 10^5^; Vivascience, Munich, Germany).

### Western blot analysis

Cell pellets were resuspended in modified RIPA buffer (50 mM Tris (pH 7.8), 150 mM NaCl, 5 mM EDTA, 15 mM MgCl_2_, 1% NP-40, 0.5% sodium deoxycholate, 1 mM DTT, protease inhibitors 1 : 100, 20 mM *N*-ethylmaleimide) and further disrupted by mechanical shearing through a 19-gauge needle. Soluble proteins were then separated by centrifugation at 4°C for 5 min. Concentration of protein was calculated using a Bio-Rad protein assay kit (Bio-Rad, Hemel Hempstead, UK). Proteins were run on 10% SDS-PAGE and blotted on nitrocellulose membrane and incubated with appropriate primary antibodies. The membranes were incubated with horseradish-peroxidase-labelled secondary antibody. The membranes were washed and developed with ECL Plus western blotting detection system (Amersham, Little Chalfont, UK). The MT1-MMP antibodies were kindly donated by Professor Murphy and used at a concentration of 1.5 *μ*g ml^−1^; 350-amino-acid isoform of DKK3 antibody (Abcam, Cambridge, UK) was used at 1 *μ*g ml^−1^.

### Reverse transcription and qRT-PCR

RNEasy (Qiagen, Crawley, UK) extraction and purification kits were used to extract total RNA from cell pellets according to the manufacturer's recommendations. SuperScript™ III first-strand synthesis system (Invitrogen) and random hexamer primers were used to reverse transcribe 2 *μ*g of total RNA according to the manufacturer's recommendations. Amplification by PCR was carried out in a final volume of 10 *μ*l containing 1.5 *μ*l of reverse-transcribed cDNA, 5 *μ*l of SYBER Green PCR master mix (Applied Biosystems, Warrington, UK) and 2 pmol of primers. ABI PRISM 7900 HT sequence detection system (Applied Biosystems) with the following PCR conditions was used: 50°C for 2 min, 95°C for 10 min, 40 cycles of 95°C for 15 s and 60°C for 1 min. As normalisation standards, GAPDH and SDH were used. Dickkopf-3 QuantiTech® primer assay (Qiagen) was used. The MT1-MMP, SDH and GAPDH primers were designed using the Primer Express software (Applied Biosystems). Sequences of the primer pairs were as follows: MT1-MMP, forward primer 5′-TGCCATGCAGAAGTTTTACGG-3′ and reverse primer 5′-TCCTTCGAACATTGGCCTTG-3′; GAPDH, forward primer 5′-GCAAATTCCATGGCACCGT-3′ and reverse primer 5′-TCGCCCCACTTGATTTTGG-3′; SDH, forward primer 5′-TGGGAACAAGAGGGCATCTG-3′ and reverse primer 5′-CCACCACTGCATCAAATTCATG-3′.

### Expression array analysis

Amino Allyl Message Amp kit (Ambion, Warrington, UK) was used according to the manufacturer's recommendation for amplification and labelling of RNA. In brief, 10 *μ*g of amplified RNA was resuspended in 6.7 *μ*l of nuclease-free water, 4.5 *μ*l of DMSO, 3.3 *μ*l of NaHCO_3_ (pH 9) and 4.5 *μ*l of Cy3 or Cy5. Labelled amplified RNA were hybridised to 22 k CRUK human cDNA expression array slides (ICR Array Facility, Surrey, UK).

### Matrigel invasion assay

A modified Boyden dual chamber was used to assess directional migration of the cells. In brief, 10 *μ*g of growth-factor-reduced Matrigel (BD Biosciences, Oxford, UK) in a total of 40 *μ*l of serum-free media was applied to the upper chamber of 8-micron-pore cell culture insert (Becton Dickinson, Oxford, UK) and allowed to set over 2 h. A total of 5 × 10^4^ cells were suspended in serum-free media and added to the upper chamber. Serum-positive media were used as chemoattractant in the lower chamber.

After a 24 h incubation period at 37°C, 5% CO_2_, the media and cells remaining in the upper chamber were removed using a cotton bud. The insert was fixed in methanol and stained using haematoxylin. The membrane was removed from the insert and the number of invading cells was quantified by counting the cells in four random per high-power fields and calculating the mean number of invading cells. All experiments were performed in triplicate.

### Scratch test

A total of 1 × 10^6^ cells were seeded onto a six-well plate and allowed to reach full confluence. The monolayer was wounded using a cocktail stick. Cells were incubated with appropriate media, as stated. Digital images were taken at times 0, 24 and 48 h. The mean area was calculated using Internet-based software Image J (http://www.rsb.info.nih.gov/ij/download.html) and compared to time 0.

### MTT cell proliferation assay

A total of 5000 cells were plated onto flat-bottomed 96-well plates and maintained overnight. Cells were incubated with 20 *μ*l of MTT (3-(4,5-dimethylthiazol-2-yl)-2,5-diphenyltetrazolium bromide) for 2 h. A volume of 200 *μ*l of DMSO was added and the absorbance measured at 492 nm.

## Results

### Reducing MT1-MMP through siRNA leads to a reduction of invasive phenotype

**Figure 1:**
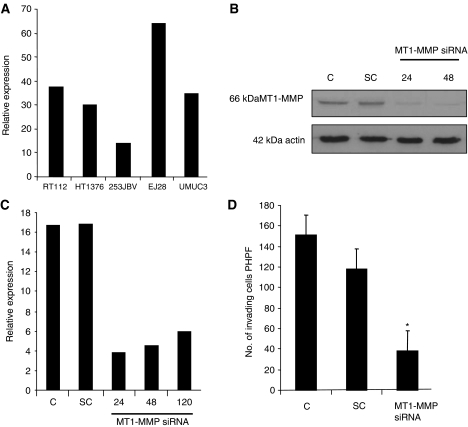
Relative expression of MT1-MMP in UCC cell lines and siRNA-mediated downregulation of MT1-MMP. (**A**) Quantitative RT-PCR of MT1-MMP mRNA. Total RNA was extracted from cultured cell lines and qRT-PCR was performed. As housekeeping genes, SDH, UBC and GAPDH were used. EJ28 cells have the highest expression levels of MT1-MMP. (**B**) Western blot of nontransfected control (C), scrambled control transfected (SC) and MT1-MMP siRNA transfected (MT1-MMP siRNA) EJ28 cells at 24 and 48 h. (**C**) Quantitative RT-PCR of MT1-MMP mRNA. (**D**) Matrigel invasion assay of nontransfected, scrambled control transfected and MT1-MMP siRNA transfected EJ28 cells. A total of 5 × 10^4^ cells were seeded and the number of cells invading through the Matrigel was counted at 24 h; ^*^P<0.05 between MT1-MMP siRNA and C or SC groups.

**Figure 2:**
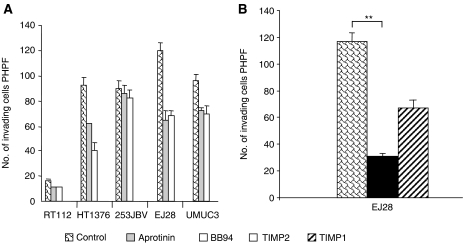
Effect of MMP and protease inhibitors on invasion of various cell lines. (**A**) A total of 5 × 10^4^ cells incubated in control media or in the presence of various inhibitors (aprotinin, BB94, TIMP1 or TIMP2) for 24 h and the number of cells invading through the Matrigel counted. (**B**) Effects of TIMP1 and TIMP2 on invasion of EJ28 cells; ^**^P<0.0005 between TIMP2 and control group.

We examined the expression of MT1-MMP mRNA in a bank of UCC cell lines, and detected the highest levels in EJ28 cells (Figure 1A), which is the most invasive cell lines, as demonstrated by Matrigel invasion assay (Figure 2A).

We confirmed that EJ28 cell invasion was dependent on MT1-MMP by siRNA transfection. Effective reduction of endogenous MT1-MMP was confirmed by western blot analysis and qRT-PCR over 120 h (Figure 1B and C) and a 70% reduction of cell invasion (*P*<0.05) was detected, when compared with wild-type and scrambled control (Figure 1D).

### Protease and MMP inhibition reduces invasive potential

Urothelial cell carcinoma cell lines were cultured with synthetic and physiological protease inhibitors, including BB94, aprotinin, TIMP1 and TIMP2, and invasion was assessed by the Matrigel assay. As shown in Figure 2A, treatment of EJ28 and HT1376 with aprotinin lead to a 45% reduction in invasiveness; treatment of EJ28 and HT1376 with BB94 to a 79% and a 55% reduction in invasiveness, respectively. Protease inhibition reduced the invasive capacity of the EJ28 cells, but had limited effect on 253JBV and UMUC3, which expressed low and moderate levels of MT1-MMP, respectively. Targeting the soluble MMPs, such as MMP2 and 13, by TIMP1 lead to a 44% reduction of invasion in EJ28 cells, whereas inhibition of all MMPs, including MT1-MMP, by TIMP2 lead to a 74% reduction (*P*<0.0005) (Figure 2B).

### Expression array identifies potential targets of MT1-MMP in EJ28 cells

To explore putative targets of MT1-MMP, we employed expression microarray to highlight differential gene transcript expression following knockdown of MT1-MMP.

**Figure 3:**
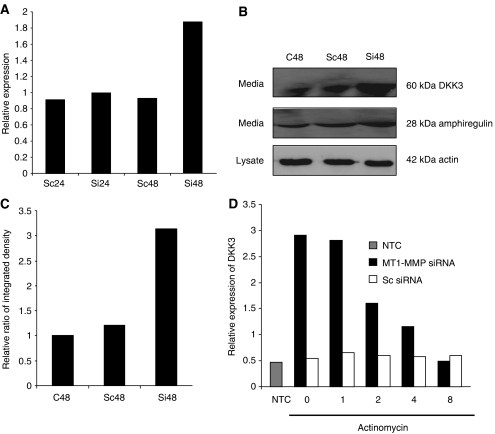
Expression of DKK3 following targeted suppression of MT1-MMP and effects of actinomycin D on DKK3 mRNA. (**A**) Quantitative RT-PCR of DKK3 mRNA following knockdown of MT1-MMP (Si) and scrambled control (SC) at 24 and 48 h expressed as a ratio of untreated controls. (**B**) Western blot analysis of DKK3 secreted in media following knockdown of MT1-MMP at 48 h compared to scrambled control (SC) transfection and wild-type EJ28 cells (C48). Actin control was used against the cell lysate, whereas amphiregulin control was used against the media. (**C**) Densitometric analysis of the western blot shown in (**B**). Image J software was used to calculate the integrated density of the inverted image and expressed as a ratio in comparison with wild-type EJ28 cells (C48) (**D**) Following the MT1-MMP knockdown and scrambled control, transfection cells were incubated with actinomycin (10 *μ*g ml^−1^) for 0, 1, 2, 4 and 8 h. The DKK3 mRNA levels were analysed by qRT-PCR and normalised against the housekeeping genes SDH and GAPDH. NTC, negative control.

A total of 70 genes were found to have a statistically different expression ratio following downregulation of MT1-MMP in EJ28 cells compared with controls. Functional analysis of these genes using gene ontology annotation through DAVID (data base for annotation, visualization and integrated discovery) revealed involvement in the regulation of cell growth and maintenance, signal transduction as well as regulation of cell cycle. Of the targets identified, our interest was drawn to DKK3, which was among the top five upregulated genes. Dickkopf-3 has been shown to act as a tumour suppressor and inhibit cell invasion (Niehrs, 2006) and is potentially negatively regulated by MT1-MMP. We confirmed this by transient knockdown of MT1-MMP at 48 h. Dickkopf-3 mRNA levels were increased 1.8-fold compared with scrambled control (Figure 3A), and an increase in secreted protein was confirmed in the culture media of MT1-MMP-suppressed EJ28 cells (Figure 3B). Densitomeric analysis of the western blot revealed that a 1.8-fold increase in mRNA levels corresponds to over 3-fold increase in protein level, as compared to a nontransfected control (Figure 3C).

### Increased levels of DKK3 following the knockdown of MT1-MMP is not as a result of mRNA stability

To determine whether knockdown of MT1-MMP in EJ28 cells affects DKK3 mRNA stability, we examined the effect of actinomycin D, an inhibitor of RNA transcription, on DKK3 mRNA levels following knockdown of MT1-MMP. In the absence of actinomycin D, the DKK3 mRNA expression was increased 2.5-fold following knockdown of MT1-MMP compared with scrambled control siRNA. In the presence of actinomycin D, DKK3 mRNA levels decreased progressively over time with minimally detectable levels of DKK3 mRNA after 8 h. The DKK3 mRNA levels at 8 h showed no statistical difference with negative control condition where no mRNA was added (*P*>0.10). These data suggest that upregulation of DKK3 is not because of enhancement of mRNA stability but as a result of transcriptional activation (Figure 3D).

### DKK3 reduces directional migration of EJ28 cells

**Figure 4:**
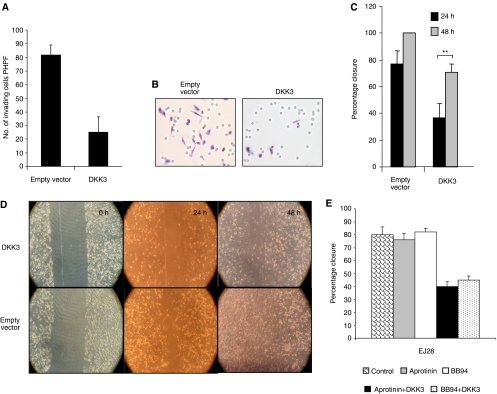
Effects of DKK3-enriched media on EJ28 directional cell migration. Empty vector or DKK3 was overexpressed in COS7 cells and serum-free conditioned media enriched in DKK3 were used in the experiment. (**A**) Matrigel invasion assay. A total of 5 × 10^4^ cells were seeded in the upper chamber and incubated with either DKK3- or empty-vector-enriched media for 24 h. The number of cells invading through the membrane was counted; ^*^P<0.005 between DKK3 and empty vector. (**B**) Photograph of Matrigel invasion assay. (**C**) Scratch test. Rate of closure of the scratch following incubation with conditioned media was measured at 24 and 48 h; ^**^P<0.01 for DKK3 at 24 and 48 h *vs* empty vector at 24 and 48 h. (**D**) Photograph of the scratch test at 24 and 48 h. (**E**) Scratch test. Rate of closure of the scratch following incubation with aprotinin, BB94 and DKK3.

To explore the effects of DKK3 on tumour cell migration, conditioned medium was enriched for DKK3. Conditioned media were derived from transient transfection of Cos-7 cells with pCS2-hDKK3, a wild-type construct, or pCDNA-T05 empty vector, and migration was assessed through Matrigel and by the scratch test assay. Addition of DKK3 conditioned media lead to a 75% reduction in the invasive capacity of wild-type EJ28 cells (*P*<0.0005) (Figure 4A and B). In keeping with this finding, DKK3-conditioned media lead to a 37 and 30% reduction in closure of scratched monolayer at 24 and 48 h, respectively, when compared with controls (*P*<0.01) (Figure 4C and D). Interestingly, addition of broad-spectrum MMP inhibitors aprotinin and BB 94 did not alter the rate of closure of scratched monolayer. However, addition of DKK3 to aprotinin and BB94 lead to a 40 and 37% reduction of the scratched monolayer, respectively (Figure 4E). We have already shown that inhibition of MMP reduces the invasive potential of UCC cell lines, as demonstrated by Matrigel invasion assay. However, these data suggest that in two-dimensional culture, MMPs may not play a significant role in would healing (scratch test), but DKK3 plays a more central role in both invasion and migration.

## Discussion

We have previously shown that among MMPs, MT1-MMP is highly expressed and associated with high grade and stage UCC (Wallard *et al*, 2006). In keeping with our previous report we have now demonstrated that MT1-MMP was expressed in all the UCC cell lines tested and high levels were detected in the more invasive cell lines. In addition, suppression of MT1-MMP using siRNA technology effectively inhibited invasion in these lines. These findings are in accordance with the accepted role in which MT1-MMP modifies the pericellular microenvironment to promote invasion and spreading of tumour cells (Itoh and Seiki, 2006).

There is emerging evidence that the cytoplasmic domain of MT1-MMP and its Src-dependent phosphorylation play a role in modulation of cell migration (Nyalendo *et al*, 2007). Sounni *et al* and Freudenberg *et al* have recently suggested that MT1-MMP has additional transcriptional activation properties, which may be involved in angiogenesis as well as in cell invasion and motility. Transcriptional activation of VEGF-A appears to be mediated via Src tyrosine kinase (Sounni *et al*, 2004), whereas induction of Smad1 is mediated via TGF-*β* signalling (Freudenberg and Chen, 2007). In invasive cell lines, we confirmed that broad-spectrum protease (aprotinin) and MMP inhibitors (BB94, TIMP1 and TIMP2), as well as direct targeting of MT1-MMP, inhibited invasion. These data support our previous observations in clinical samples and suggest that MT1-MMP is a critical MMP promoting UCC cell invasion. The inhibition of invasion upon targeting MT1-MMP by TIMP2 and the less striking effect of TIMP1 on EJ28 cells lead us to explore potential downstream targets of MT1-MMP contributing to EJ28 cell invasion. To further indirectly support the notion that factors other than activation of MMP2 play a role in invasion of EJ28 cells, profiling of UCC cell lines showed that EJ28 cells express one of the highest levels of MT1-MMP, whereas their MMP2 expression levels are low compared with other cell lines (Supplementary data).

Using a corneal myofibroblasts model, Ottino *et al* (2005) have also shown that activation of MT1-MMP by inflammatory mediator platelet activation factor does not result in increased MMP2 activity, suggesting an alternative pathway for extracellular matrix (ECM) remodelling in the cornea.

We explored potential MT1-MMP-related targets and used expression array technology to identify gene alterations following suppression of MT1-MMP in EJ28 cells. Of the targets identified, our interest was drawn to DKK3, which is a tumour suppressor and inhibits cell invasion (Niehrs, 2006).

In this study, expression of DKK3 was induced following downregulation of MT1-MMP and elevated levels of the protein were detected in the growth medium of MT1-MMP-repressed cells. Overexpression of DKK3 in conditioned media lead to significant reduction of invasion in UCC. Expression levels of DKK3 mRNA decreased progressively over time following incubation with actinomycin D, suggesting transcriptional regulation of DKK3 by MT1-MMP rather than mRNA stability. These results support studies that point toward a role in which MT1-MMP controls the transcriptional regulation of a number of genes important in the tumour progression machinery. As far as we are aware, modulation of DKK3 by MT1-MMP has not been reported previously. Whether this transcriptional regulation is a direct or an indirect effect remains to be elucidated. One possible explanation for this transcriptional effect observed could be internalisation of MT1-MMP and, in particular, localisation of the cytoplasmic domain in the nucleus and direct effect on transcription (Ip *et al*, 2007). This mechanism has previously been implicated in the regulation of transcription by the ErbB4 and Notch receptors, which require proteolytic cleavage and nuclear translocation of their cytoplasmic domains. However, this may represent a rather unique case and it is more commonly accepted that degradation of the ECM by MT1-MMP modulates signals generated by integrin–ECM interactions, which consequently affect gene transcription. A further consideration would be transcriptional regulation as an indirect result of activation of other pathways.

### Future work

There remain many unanswered questions regarding the exact mechanism of modulation of DKK3 by MT1-MMP. Indeed, it would be very interesting to pose the question that in view of the fact that DKK3 is considered to be a tumour suppressor gene whether it can also regulate MT1-MMP, suggesting a feed back loop. To establish the mechanism of the modulation of DKK3 by MT1-MMP, we plan to use various mutants of MT1-MMP in our knockdown system to investigate the contribution made by various elements of MT1-MMP protein.

In conclusion, we have shown that MT1-MMP is essential for the invasion of UCC cell lines, and involves hitherto unreported mechanisms. Membrane type-1-MMP appears to transcriptionally modulate DKK3 levels, which is a potent inhibitor of invasion.
