# Supplementary material for: MT1-MMP regulates urothelial cell invasion via transcriptional regulation of Dickkopf-3
Source: Br J Cancer. 2008 Jul 29;99(4):663–9. doi: 10.1038/sj.bjc.6604513 (PMC2527828; doi:10.1038/sj.bjc.6604513)
Supplement: Supplementary data [file 6604513x1.ppt]

## Slide 1
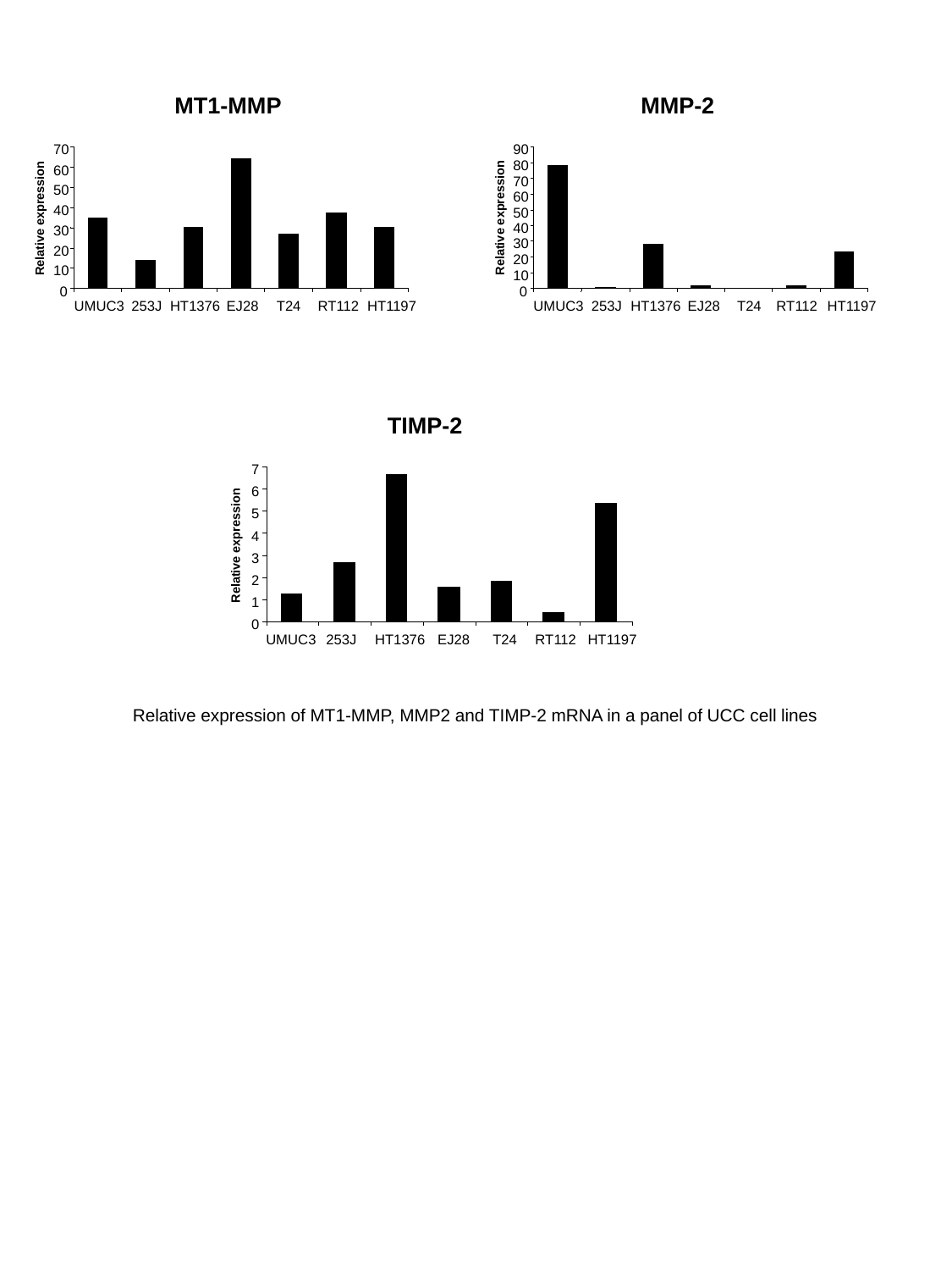

MMP-2
90
80
70
60
50
Relative expression
40
30
20
10
0
UMUC3
253J
HT1376
EJ28
T24
RT112
HT1197
MT1-MMP
70
60
50
40
Relative expression
30
20
10
0
UMUC3
253J
HT1376
EJ28
T24
RT112
HT1197
TIMP-2
7
6
5
4
Relative expression
3
2
1
0
UMUC3
253J
HT1376
EJ28
T24
RT112
HT1197
Relative expression of MT1-MMP, MMP2 and TIMP-2 mRNA in a panel of UCC cell lines
